# Laparoscopically excised retroperitoneal presacral Schwannoma: atypical pre and postoperative manifestations – case report

**DOI:** 10.1186/s12893-019-0611-8

**Published:** 2019-10-22

**Authors:** Bárbara Justo Carvalho, Kayo Augusto de Almeida Medeiros, Diego Ramos Martines, Fernanda Nii, Leonardo Zumerkorn Pipek, Gustavo Heluani Antunes de Mesquita, Luiz Augusto Carneiro D’Albuquerque, Alberto Meyer, Wellington Andraus

**Affiliations:** 1 Faculdade de Medicina da Universidade de São Paulo, Avenida Doutor Arnaldo, 455, São Paulo, SP 01246-903 Brazil; 2 Hospital das Clínicas da Faculdade de Medicina da Universidade de São Paulo, Avenida Doutor Enéas Carvalho de Aguiar, 155, São Paulo, SP 05403-000 Brazil; 3 Samaritano Hospital, Rua Conselheiro Brotero,1486, São Paulo, SP 01232-010 Brazil

**Keywords:** Neurilemomma, Laparoscopy, Postoperative complications, Papillary carcinoma, Multiple endocrine Neoplasia

## Abstract

**Background:**

We are a reporting a rare case of retroperitoneal schwanomma with atypical pre and postoperative manifestations. Retroperitoneal schwannomas are rare tumors that are difficult to preoperatively diagnose.

**Case presentation:**

This is a case report of a male patient, 41 years old, with symptoms of hipogastric and lower right member pain, as well as a history of a papilliferous thyroid tumor. Computerized tomography exams were inconclusive, showing a mass in the presacral region with dimensions of 4.4 × 3.9 × 3.4 cm. Removal was carried out by laparoscopic surgery, with self-limited postoperative complications. Diagnosis was carried out by anatomopathological examination, and syndromic hypotheses were discarded.

**Conclusions:**

The postoperative complications of schwanomma are little reported in the literature. In the simultaneous occurrence of schwanomma and other endocrine tumors, further studies are warranted to better differentiate the cases that need investigation of syndromic causes.

## Background

Schwannomas are mostly benign tumors derived from Schwann cells in the peripheral and cranial nerves. Retroperitoneal schwannomas are rare, accounting for 0.5 to 3% of all schwannomas, and 1% of all retroperitoneal neoplasias [1]. They occur principally in the third to fifth decade of life [2]. Such tumors are generally located adjacent to the peripheral nerve of origin, and are enclosed by an epithelial capsule, with non-specific symptomology dependent on the location and size of the lesion.

Among the differential diagnoses, there are paragangliomas, pheochromocytomas, osteoblastomas and histiocystic sarcomas [1], with imaging and anatomopathological examinations necessary to reach a definitive diagnosis [2]. In the rare cases of malignant tumors, early diagnosis is important due to the low rate of response to chemotherapy and radiotherapy and the risk of invasion of adjacent structures [3]. We are a reporting a rare case of retroperitoneal schwanomma with atypical pre and postoperative manifestations in a patient with a history of papilliferous thyroid tumor.

## Case presentation

**Fig. 1:**
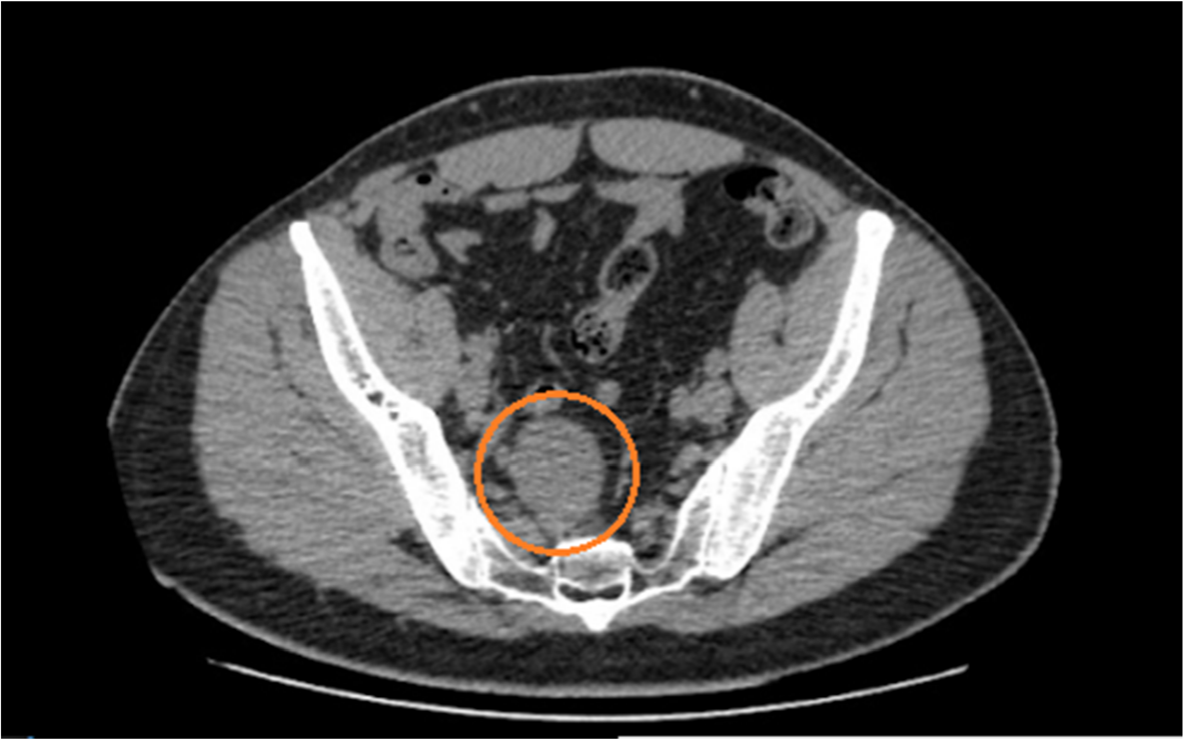
CT of the transversal plane of the presacral region. Note the schwannoma (red circle), with dimensions of 4.4 × 3.9 × 3.4 cm

**Fig. 2:**
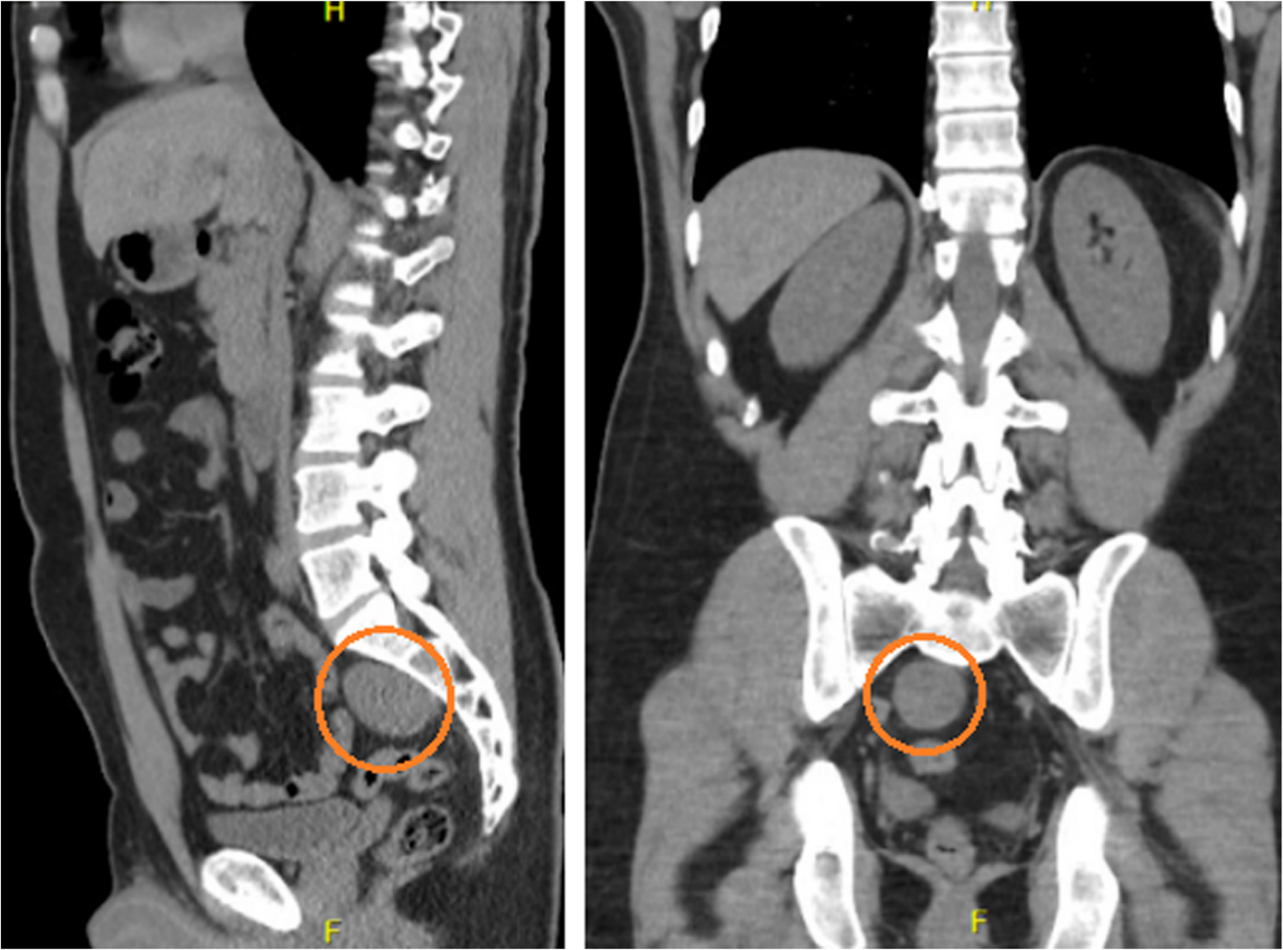
CT of the sagittal and coronal planes of the presacral region, respectively. Note the schwannoma (red circle), with dimensions of 4.4 × 3.9 × 3.4 cm

A 41 year old male presented at the emergency room reporting abdominal pain in the hypogastric region and in the lower right limb with neurogenic characteristics (paresthesia and burning mainly in the L2 dermatome) one week prior to admission. The patient had a history of papilliferous tumor of the thyroid, resected two years previously without need for complementary therapy. The patient underwent computerized tomography (CT) of the abdomen (Figs. 1 and 2), which revealed a hypodense and circumscript nodular formation, located in the right median presacral region, at the level of vertebrae S1 and S2, measuring approximately 4.4 × 3.9 × 3.4 cm. There were no other abdominal abnormalities. The decision to perform an excision surgery was based on the symptoms (associated with the topography of the tumor) and the need of diagnosis since the CT was inconclusive. There was no suspicious diagnosis related to the previous tumor.

**Fig. 3:**
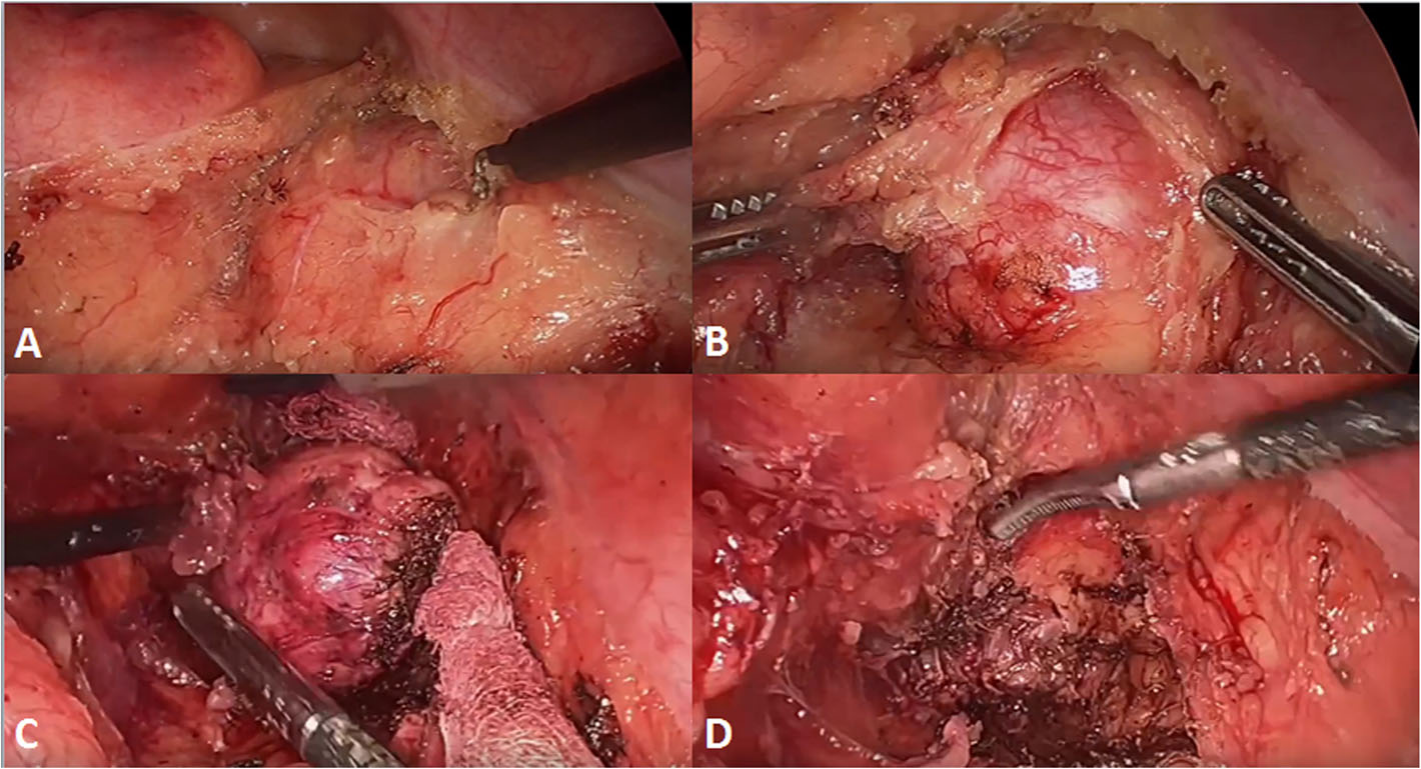
Intraoperative images. **a**: The tumor in retroperitoneal space before dissection. **b**: Capsule dissection. **c**: Total removal of capsule. **d**: Cavity after schwanomma removal

The tumor found in laparoscopic surgery was a hardened nodular mass with a yellow surface and a elastic consistency. Its capsule was thickened by a significant adipose tissue layer firmly adhered to its surroundings. A colon liberation and meticulous dissection was performed in order to expose the tumor and to remove the adherences without harming important structures as the iliac vessels and the inferior hypogastric plexus (Fig. 3).

**Fig. 4:**
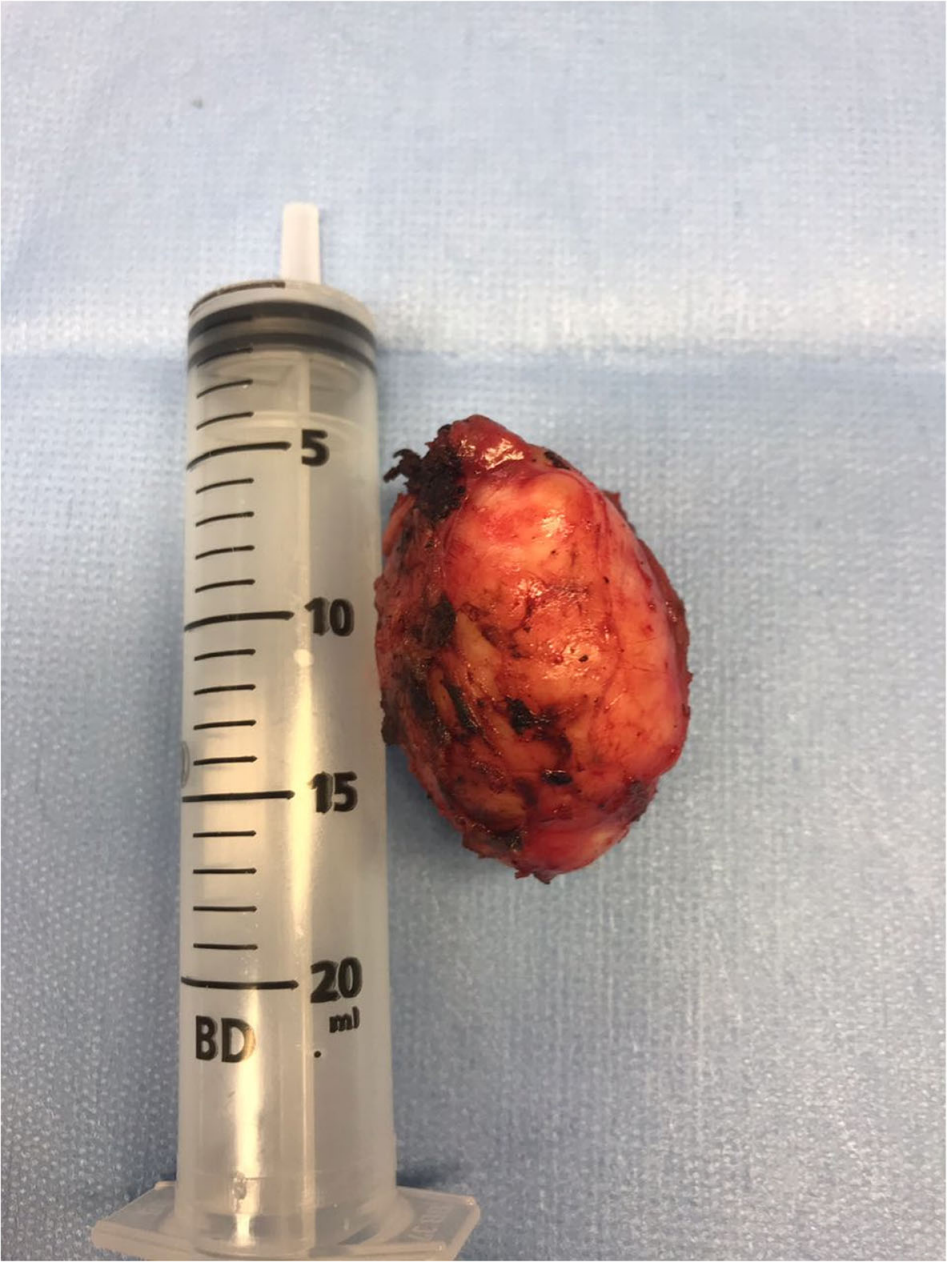
The tumor

The lesion was completely removed, without signs of bleeding or remaning lesions. Histopathological examination revealed dimensions of 5.0 × 3.5 × 2.6 cm (Fig. 4). The surgery lasted 260 min and was performed through 4 incisions (right lumbar region, left and right iliac regions and infra-umbilical). The discharge was made after less than 24 h of hospitalization. The patient reported alteration in intestinal routine, retrograde ejaculation and pain in the lower right limb in the immediate postoperative phase, with spontaneous resolution after approximately one month. The patient was followed up for 4 months and there were no further complications.

The microscopy showed cells with elongated nuclei, arranged in vertices or in a wave pattern, next to areas with few cells (Antoni B areas) with edema, some containing microcysts and signs of previous hemorrhage. In the more populated areas (Antoni A), structures were characterized by nuclei present in two rows including almost homogeneous anuclear matter.

The immunohistochemical examination, carried out with antigen retrieval with moist heat (PT Link-Dako), revealed that the tumor was a schwannoma, positive for protein S-100, GFAP, AE1 and AE3 (these last two in rare cells) and negative for CD34. KI-67 was positive in less than 1% of cells, characterizing a benign schwanomma. Immunohistochemical investigation was carried out for melanocytic schwannoma for suspected Carney Complex [4], which returned a negative result (HMB45, Melan A, negative smooth muscle actin, positive vimentin).

## Discussion and conclusion

Benign schwannomas are mostly found in the cephalocervical region (44.8%) and in limbs (32.6%), and are rarely situated in the retroperitoneal (0.7%) [5]. Retroperitoneal schwannomas are mostly asymptomatic and unintentionally found by imaging exams [6]. When symptoms are present, the most common are distension or abdominal pain, more uncommon are dysesthesia, lower lumbar pain, urinary incontinence and alteration of intestinal habits [3, 7]. The literature does not frequently report pain in lower limbs.

Given that the tumor was smaller than the mean reported in the literature (13,7 cm at diagnosis by Li et al. [3]), it is unusual that this tumor presented clinical symptoms leading to diagnosis when the majority are asymptomatic.

Despite there is still controversy about the best approach for this type of surgery, laparoscopic surgery was considered appropriate for the treatment of this tumor. Beyond the well-known general advantages, such as shorter postoperative recovery, laparoscopic surgery has been recognized in the literature as a feasible and efficient method in the treatment of this kind of tumor [8–10] and permits the magnification of the image and the best view of the surgical field [5]. This is relevant for a delicate procedure such as this due to retroperitoneal schwannomas being close to nerves and important vascular structures. However, in recurrent schwannomas and those at high risk of hemorrhage, tumor rupture and malignancy, open surgery should be considered the best option, mainly if combined with inexperience of the surgeon [11].

Our case had a long operative time when compared to other literature cases of retroperitoneal schwanommas [9, 11]. We assumed that this happened due to the significant difficulty to access the presacral retroperitoneal region, the need of meticulous dissection to preserve important structures and the peculiarity of a thickened and adhered capsule as mentioned before.

The postoperative complications in this type of surgical procedure are little reported in the literature, with recurrence the most common (5–10%), associated with the rupture or partial excision of the tumor [11]. In our case, the complications indicated dysfunction of the autonomous nervous system due to manipulation of the hypogastric plexus. Retrograde ejaculation was reported only by Nedelcu et al. [12] in a study of the laparoscopic excision of nine retrorectal tumors, among them four schwannomas. Alterations of intestinal habit have not been reported as postoperative complications. Although the case had these complications, we believe that a open surgery wouldn’t be a better option since it involves even more manipulation of the structures, which could lead to even more complex and long-lasting injuries in the removal of a tumor origined in nerves.

The anatomopathological examination presented a typical schwannoma. Diagnosis was made on this basis, since the computerized tomography did not reveal any signs (such as hemorrhage, necrosis or liquefaction) [3] that could differentiate the lesion from lymphomas or cystic formations. This highlights the difficulty of preoperative diagnosis [2, 3].

The history of resection of the papilliferous thyroid tumor led to suspicions of possible systemic causes. An immunohistochemical analysis for melanocytic schwannoma was carried out because, like the presence of thyroid neoplasia, it is one of the main criteria for diagnosis of Carney Complex [4]. This autosomatic dominant hereditary illness is occasioned by loss of function of the tumoral supressor gene PRKAR1A. The negative result, together with the absence of other main and supplementary criteria for diagnosis of the disease, led us to discount Carney Complex.

Other syndromes with schwannoma among its typical lesions, such as neurofibromatosis type 2 and schwannomatosis were also discounted due to the absence of other criteria that confirm the diagnosis. Although there is a criterion that suggests the necessity of genetic testing from a positive biopsy for schwannomatosis, the absence of multiple tumors and suspect signs in computerized tomography meant that the condition was improbable.

Retroperitoneal schwannomas are tumors that are unlikely to be preoperatively diagnosed, and that require surgical experience in laparoscopic excision. Their postoperative complications are little reported in the literature.

The appearance of schwannomas in patients with a history of other tumors can be coincidental but in some cases (especially endocrine tumors), it can be indicative of syndromic causes. In our case, we did not find that a deep investigation of syndromic causes should be a concern, but further studies are warranted to better differentiate the cases that need the investigation.

## Data Availability

All data produced and obtained is available within the manuscript.
